# High‐Throughput Fabrication of Flexible and Transparent All‐Carbon Nanotube Electronics

**DOI:** 10.1002/advs.201700965

**Published:** 2018-02-20

**Authors:** Yong‐Yang Chen, Yun Sun, Qian‐Bing Zhu, Bing‐Wei Wang, Xin Yan, Song Qiu, Qing‐Wen Li, Peng‐Xiang Hou, Chang Liu, Dong‐Ming Sun, Hui‐Ming Cheng

**Affiliations:** ^1^ College of Information Science and Engineering Northeastern University 3‐11 Wenhua Road Shenyang 110819 China; ^2^ Shenyang National Laboratory for Materials Science Institute of Metal Research Chinese Academy of Sciences 72 Wenhua Road Shenyang 110016 China; ^3^ School of Material Science and Engineering University of Science and Technology of China 96 Jinzhai Road Hefei 230026 China; ^4^ University of Chinese Academy of Sciences 19 A Yuquan Road Beijing 100049 China; ^5^ Suzhou Institute of Nano‐Tech and Nano‐Bionics Chinese Academy of Sciences 398 Ruoshui Road Suzhou 215123 China; ^6^ Tsinghua‐Berkeley Shenzhen Institute Tsinghua University 1001 Xueyuan Road Shenzhen 518055 China

**Keywords:** carbon nanotubes, flexible electronics, photosensitive dry films, thin‐film transistors, transparent electronics

## Abstract

This study reports a simple and effective technique for the high‐throughput fabrication of flexible all‐carbon nanotube (CNT) electronics using a photosensitive dry film instead of traditional liquid photoresists. A 10 in. sized photosensitive dry film is laminated onto a flexible substrate by a roll‐to‐roll technology, and a 5 µm pattern resolution of the resulting CNT films is achieved for the construction of flexible and transparent all‐CNT thin‐film transistors (TFTs) and integrated circuits. The fabricated TFTs exhibit a desirable electrical performance including an on–off current ratio of more than 10^5^, a carrier mobility of 33 cm^2^ V^−1^ s^−1^, and a small hysteresis. The standard deviations of on‐current and mobility are, respectively, 5% and 2% of the average value, demonstrating the excellent reproducibility and uniformity of the devices, which allows constructing a large noise margin inverter circuit with a voltage gain of 30. This study indicates that a photosensitive dry film is very promising for the low‐cost, fast, reliable, and scalable fabrication of flexible and transparent CNT‐based integrated circuits, and opens up opportunities for future high‐throughput CNT‐based printed electronics.

Carbon nanotubes (CNTs) have been considered one of the most promising candidates for flexible and transparent carbon‐based electronic devices due to their outstanding mechanical flexibility, stretchability, and electrical conductivity.^1^, ^2^, ^3^, ^4^, ^5^, ^6^, ^7^, ^8^, ^9^, ^10^ A reliable fabrication and patterning technology for mass production is the key to taking full advantage of them in practical applications. Notable progress has been made on the large‐scale fabrication of CNT thin films in recent years using floating catalyst chemical vapor deposition (CVD)^1^, ^2^, ^11^ or solution‐processed deposition methods.^12^, ^13^, ^14^ However, due to the lack of techniques that allow the deposition of liquid photoresists on an extremely large scale, CNT‐based electronic devices have been limited to the wafer‐scale^12^, ^15^, ^16^ or medium‐scale integration level.^17^, ^18^ Printing technology, such as inkjet printing,^19^, ^20^, ^21^, ^22^, ^23^, ^24^ screen printing,^25^, ^26^, ^27^, ^28^ and gravure printing,^29^, ^30^ in manufacturing electronics has drawn tremendous interest during the past few decades. Compared with traditional multi‐step photolithography, printing is a cost‐effective and scalable technology with high throughput and high compatibility to transferring and fabricating large‐scale nanocarbon electronics like the roll‐to‐roll printing technique,^31^, ^32^ which provides an important way to achieve the mass production of large‐scale flexible electronics at extremely low cost. Unfortunately, low pattern resolution is an inevitable problem that impedes the scalability of roll‐to‐roll printing for CNT‐based flexible electronics in order to satisfy the demands of versatile applications. Therefore, exploring an effective technique for patterning CNT thin films on a large scale with a high resolution is critical for the mass production of CNT‐based flexible and transparent electronic devices.

A photosensitive dry film is a polymer compound, which can be polymerized when exposed to an ultraviolet (UV) irradiation to form a stable film attached to the surface of substrate, achieving the function of patterning and etching. Although it was originally developed for the fabrication of large‐scale printed circuit boards,^33^, ^34^, ^35^ it has also been used in electroplating molds, microfluidic channels,^36^, ^37^, ^38^ and microscope‐based maskless micropatterning.^39^ The original method for the rapid prototyping of printed circuit boards (with a resolution of 100 µm) has several inherent advantages compared to conventional photoresists, including low cost, good conformability, excellent adhesion to any substrate, good flatness, uniform thickness, and no need for liquid handling during the lamination process.^37^ In addition to its own flexibility and film nature, it is compatible with roll‐to‐roll printing technology, showing great potential in the mass production of CNT‐based flexible and transparent electronic devices.

Here, we propose a simple and effective technique to fabricate flexible all‐CNT electronics using photosensitive dry films instead of traditional liquid photoresists. Through systematic parameter optimization, a 10 in. sized photosensitive dry film was efficiently laminated onto a flexible substrate by a roll‐to‐roll technique, and a 5 µm patterning resolution of CNT films was achieved for the construction of flexible and transparent all‐CNT thin‐film transistors (TFTs) and integrated circuits. The all‐CNT TFTs we fabricated exhibited good electrical performance and excellent reproducibility and uniformity, which allowed us to construct an inverter circuit with a large noise margin.

A photosensitive dry film (DuPont Riston MX9010) is a kind of negative photoresist consisting of photopolymers, which becomes insoluble after exposure to UV radiation. After removing the protective film coating on the photosensitive dry film, the photosensitive layer, consisting of a host resin and a photoinitiator, was laminated onto a flexible substrate. **Figure** **1**a illustrates the lamination and patterning process of the film on a polyethylene naphthalate (PEN) substrate with a thickness of 125 µm. The film was laminated onto the substrate by a roll‐to‐roll technique under optimized conditions of speed, pressure, and temperature (see the Experimental Section for details) to produce a strong adhesion. After exposure and developing, the film formed fine‐featured patterns on the substrate. Figure 1b,c shows photographs of a 10 in. (A5 paper size) device pattern on a PEN substrate after lamination and after exposure, respectively (see the videos in the Supporting Information). Even larger area patterns can be produced by this method using a larger film. The good uniformity achieved indicates that this roll‐to‐roll lamination technique is promising for the large‐scale fabrication of flexible electronics.

**Figure 1:**
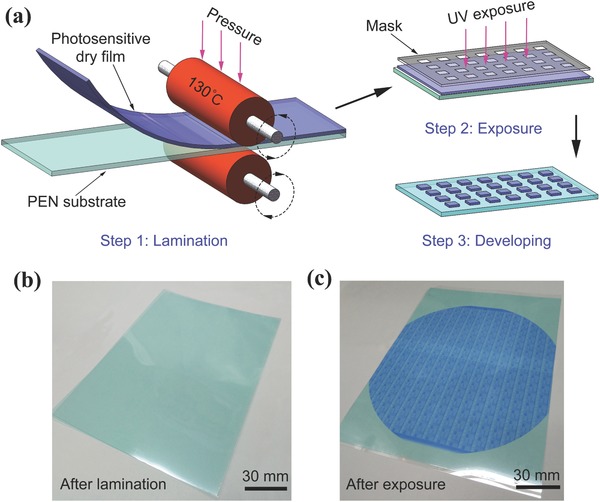
Device fabrication based on a photosensitive dry film. a) Schematic of the lamination and patterning processes of a dry film on a flexible substrate: step 1, a dry film is laminated onto a PEN substrate by a roll‐to‐roll technique under optimized temperature and pressure conditions; step 2, UV exposure of the dry film through a mask pattern; and step 3, the pattern is formed after a developing process. b,c) Photographs of real devices in A5 paper size showing views after lamination and after exposure.

The experimental conditions including exposure energy and postbaking temperature have a significant influence on the morphology of the patterns, because these conditions greatly affect the activation and stabilization of the polymerization of the photopolymers on the photosensitive dry film. A series of experiments were carried out to avoid either under or over exposure and baking that result in a loss of defined accuracy (Figure S1, Supporting Information). **Figure** **2**a shows a large area and uniform patterned image under the optimized conditions (see the Experimental Section for details). As shown in Figure 2b–d, various patterns including three‐quarter/full rings and lines of different sizes exhibit isotropic properties, allowing us to construct a complex morphology by this technique. It was found that the reproducible optimum pattern resolution of photosensitive dry films is up to 5 µm, which should be good enough for the fabrication of flexible macroelectronics based on various semiconductor materials including the CNT film.

**Figure 2:**
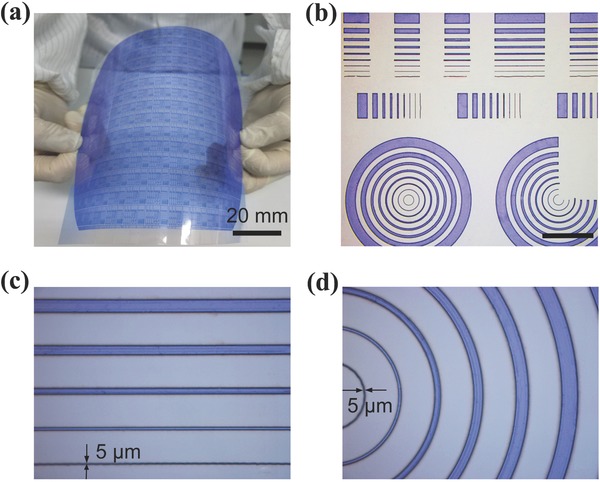
Patterning of the photosensitive dry film. a) Photograph of a large area and uniform patterning image of a flexible and transparent PEN substrate. b–d) Optical microscopy images of various patterns including rings and lines of different sizes to demonstrate the resolution of the fabrication process.

We fabricated flexible and transparent top‐gate all‐CNT TFTs on a PEN substrate using this photosensitive dry film patterning technique (Figure S2, Supporting Information). **Figure** **3**a shows that both the active channel layer and the passive elements of the gate, source, drain electrodes, and interconnections are made up of CNT films, and the dielectric is a 600‐nm‐thick polymethyl methacrylate (PMMA) layer for the effective suppression of the device hysteresis (Figure S3, Supporting Information). Figure 3b,c shows optical microscopy images of the fabricated TFT and arrays. Both the channel length (*L*_ch_) and channel width (*W*_ch_) of the TFTs are 100 µm. Figure 3d–f shows the scanning electron microscopy (SEM) images of the TFT, CNT electrode, and CNT channel. There are obvious differences in the morphology between the CNT electrode film and the CNT channel film. The CNT films for electrodes were synthesized by floating‐catalyst CVD and transferred from a membrane filter onto the PEN substrate through a roll‐to‐roll printing technique (see the Experimental Section for details). Semiconducting CNT films for the channels were deposited by immersing the substrate in a high‐purity semiconducting CNT suspension, and the density of the CNT film can be adjusted by tuning the deposition time (Figure S4, Supporting Information). The sheet resistance of the CNT electrodes and interconnections is 359 Ω ▫^−1^ and the contact resistance between the electrode and the channel is 330 Ω (Figure S5, Supporting Information). Figure 3g,h illustrates the flexibility and transparency of the fabricated devices. The transmittance of the all‐CNT TFT devices is ≈81%, which is about 10% lower than that of the bare PEN.

**Figure 3:**
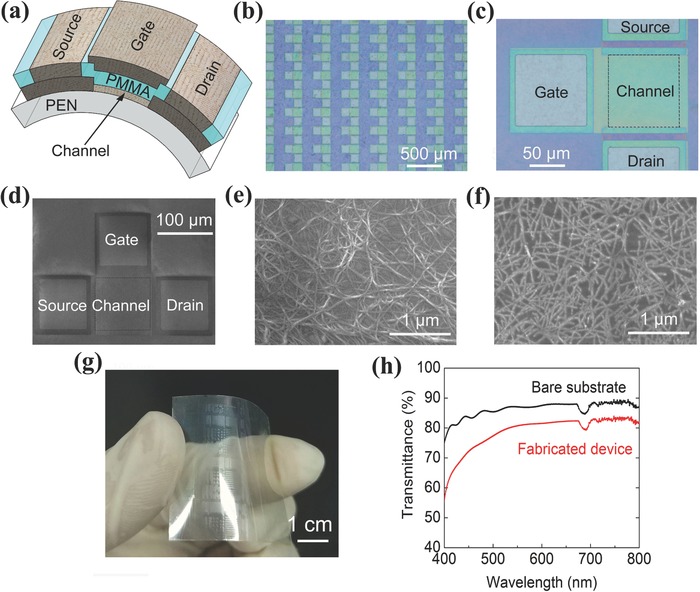
Flexible and transparent all‐CNT TFTs. a) Schematic of a top‐gate TFT on a PEN substrate, where the source, drain, and gate electrodes consist of a thick CNT film, the channel is a semiconducting CNT film, and the gate dielectric is a PMMA layer. b) Optical microscopy image of a top‐gate all‐CNT TFT array. c) Magnified image showing the structures of the source, drain, gate, and channel patterns. d–f) SEM images of a top‐gate all‐CNT TFT, a CNT electrode, and a CNT channel. g) Photograph of an all‐carbon device fabricated on a flexible PEN substrate. h) Optical transmittance of a bare substrate (black line) and the device fabricated on the substrate (red line).

**Figure** **4**a gives the typical transfer characteristics of a top‐gate all‐CNT TFT at a source/drain voltage (*V*_DS_) of −1 V. The device showed p‐type characteristics with an on–off current ratio of >10^5^ and a hysteresis of 4 V when the source/gate voltage (*V*_GS_) ranges from −30 to 30 V. The carrier mobility (*µ*) of the devices fabricated was high as 33 cm^2^ V^−1^ s^−1^, which was evaluated using the standard formula *µ* = (*L*_ch_/*W*_ch_)(1/*C*)(1/*V*_DS_)(d*I*_DS_/d*V*_GS_). *C* is the gate capacitance calculated using a parallel plate model as ε/*t*_ox_, where *t*_ox_ and ε are the thickness and dielectric constant, respectively, of the gate insulator.^1^ Forty‐five all‐CNT TFTs were measured, exhibiting almost the same curve shapes for the transfer characteristics, as shown in Figure 4b. Figure 4c shows the corresponding distributions of on‐current, off‐current, and carrier mobility of 45 devices. Note that the standard deviations of on‐current and mobility are only 5% and 2% of the average value, respectively, which represents great progress in the reproducibility and uniformity of a device property than given in a previous report,^2^ where the values were 32% and 28%, respectively. Figure 4d shows an ohmic behavior in the linear region of the output characteristics of the same device in Figure 4a, suggesting a formation of good ohmic contacts between the CNT electrodes and CNT channel.

**Figure 4:**
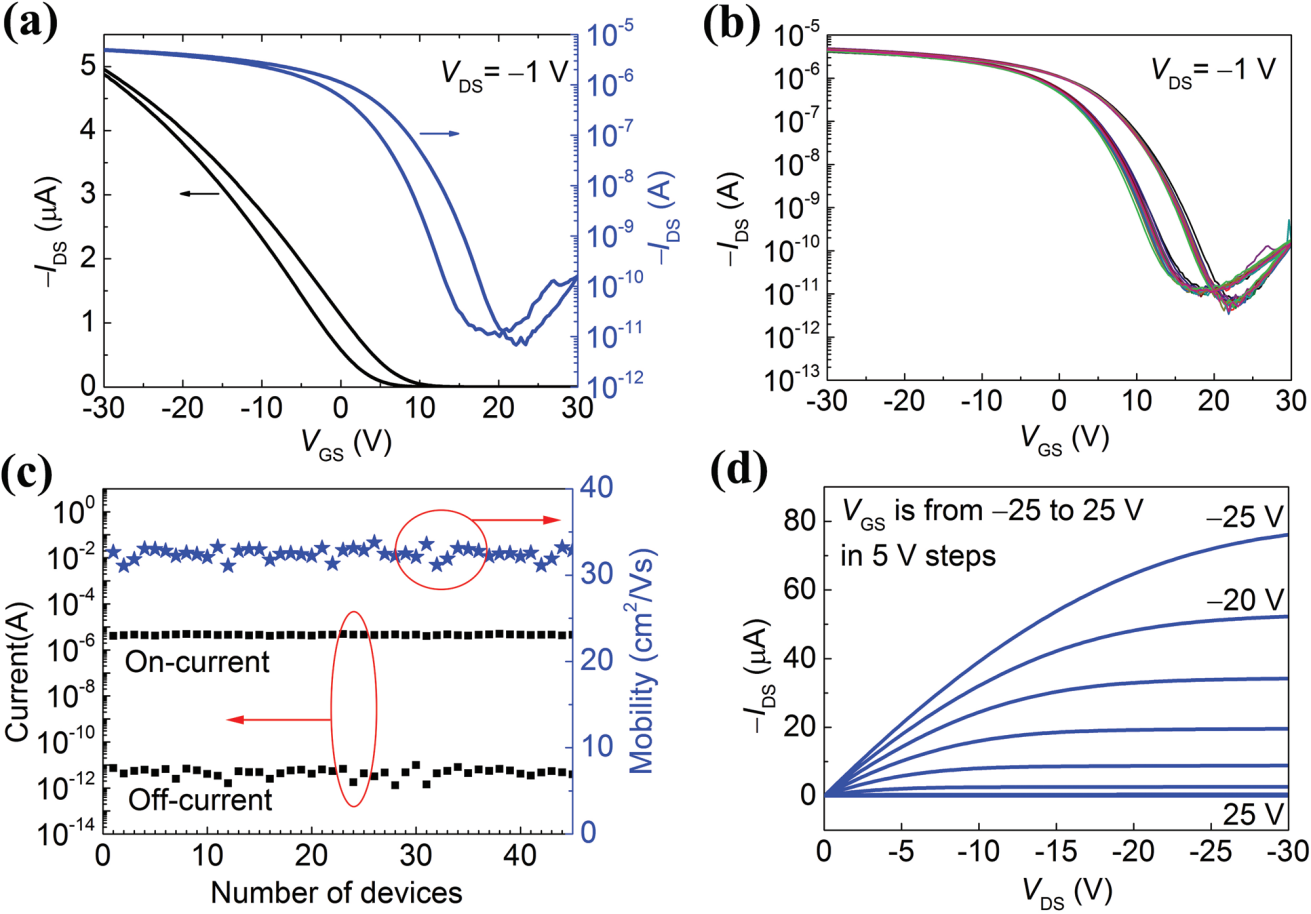
Electrical performance of all‐CNT TFTs. a) Typical transfer (*I*_DS_−*V*_GS_) characteristics with linear and logarithmic scales for the current at *V*_DS_ = −1 V. *W*_ch_ = *L*_ch_ = 100 µm. b) Transfer characteristics of 45 TFTs demonstrating good uniformity. c) Corresponding distributions of on‐current, off‐current, and carrier mobility of 45 devices. The standard deviations of on‐current and mobility are 5% and 2% of the average value, respectively. d) Output (*I*_DS_−*V*_DS_) characteristics of the same device in (a) exhibiting saturation behavior.

The good reproducibility and uniformity of the fabricated TFTs allow us to construct logic integrated circuits. Here, we demonstrate flexible and transparent all‐CNT inverters using the same structural parameters of CNT electrodes and PMMA insulators as those in all‐CNT TFTs. The load for the logic gates is a gate–source‐shorted all‐CNT TFT. It is noticed that the TFTs operate in the normally on condition, as shown in Figure 4a, i.e., the devices will not shut off completely when a zero gate voltage is applied. As shown in **Figure** **5**a, the channel of the load TFTs is designed to be a stripe to adjust the threshold of the inverter in order to obtain the normally on inverter with the excellent performance based on the output characteristics of the driver TFT and load TFT in a same all‐CNT inverter (Figure S6, Supporting Information), thus the equivalent channel width of the load and driver TFTs are 50 and 100 µm, respectively. Figure 5b shows the excellent input–output characteristics of the all‐CNT inverter. In spite of the high operation voltage, it can be decreased effectively by using other kinds of thinner gate dielectrics (Figure S7, Supporting Information). The large area of the eye pattern in the folded transfer curve implies a large noise margin for logic operation. Moreover, the total noise margin is around 0.8 on the basis of formula (*V*_OH_ − *V*_IH_ + *V*_IL_ − *V*_OL_)/*V*_DD_ (Figure S8, Supporting Information) and high enough to allow us to construct logic integrated circuits.^18^ The noise margin decreases due to the existing hysteresis of the present TFTs even though the hysteresis is small. The inverters exhibit full rail‐to‐rail input–output characteristics (Figure 5c) and a high voltage gain of 30 (Figure 5d) under a wide range of operating voltages (*V*_DD_) from −40 to −15 V. In addition, the transfer characteristics of the same all‐CNT TFT and inverter at various bending radius almost keep constant indicating the good stability and flexibility (Figure S9, Supporting Information). Good uniformity of electrical performance was demonstrated based on the input–output characteristics of 14 all‐CNT inverters (Figure S10a, Supporting Information). Note that the hysteresis could adversely influence the repeatable and robust operation of logic circuits. The hysteresis of each inverter increases with the sweep amplitude of *V*_GS_ (Figure S10b–f, Supporting Information).

**Figure 5:**
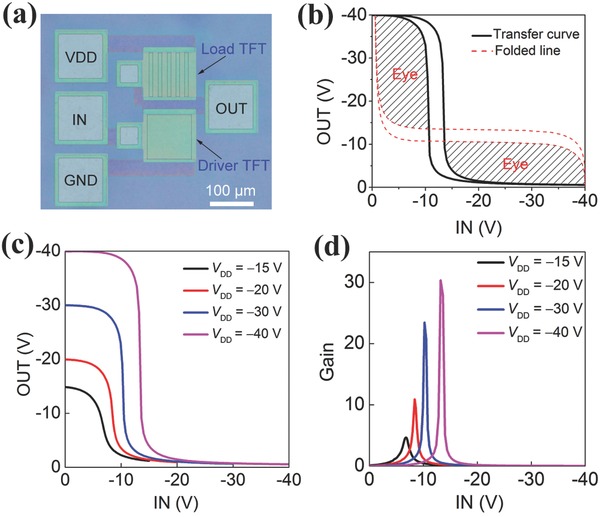
An all‐CNT inverter. a) Optical microscopy image of a p‐type metal‐oxide‐semiconductor inverter with different channel widths for load and driver TFTs. b) Input–output characteristics with hysteresis of the inverter. The input voltage swept from −40 to 0 V and back to −40 V. Two apparent eye patterns remain in the folded transfer curves demonstrating a large noise margin for logic operation. c,d) Input–output and corresponding gain characteristics of the all‐CNT inverter at various *V*_DS_ values ranging from −40 to −15 V.

For the first time we have developed a simple and effective technique for the fabrication of flexible and transparent all‐CNT TFTs and integrated circuits by using a photosensitive dry film. Ten inch sized flexible devices with a 5 µm patterning resolution have been achieved by a roll‐to‐roll technology. The all‐CNT TFTs fabricated by this technique exhibit good electrical performance including an on–off current ratio of >10^5^, a carrier mobility of 33 cm^2^ V^−1^ s^−1^, and a good uniformity, allowing us to construct high‐performance integrated circuits. These results indicate that the photosensitive dry film is very promising for the low‐cost, fast, reliable, and scalable fabrication of flexible and transparent CNT‐based integrated circuits.

## Experimental Section

*Preparation of CNT Films for Electrodes*: The CNTs were synthesized at 1100 °C by a floating‐catalyst CVD method using ethylene as the carbon source and ferrocene as the catalyst precursor.^40^ The CNT network was collected by filtering through a membrane filter (Millipore) of cellulose acetate mixed with nitrocellulose at room temperature, whose density can be precisely controlled by adjusting the collection time to make it suitable for use as electrodes in all‐CNT devices.

*Preparation of Semiconducting CNT Films for Channel Material*: The semiconducting CNTs were sorted from bulk CNTs purchased from Carbon Solution Inc. A dispersant, 9‐(1‐octylonoyl)‐9H‐carbazole‐2,7‐diyl (PCz), was prepared by Suzuki polycondensation.^12^ Dispersant PCz (100 mg) and single wall CNTs (100 mg) were mixed in xylene (100 mL). The solutions were ultrasonically stirred using a top‐tip dispergator (Sonics VC500) for 30 min at an amplitude level of 30% and then centrifuged at 45 000 g for 1 h to remove bundles and undispersed materials. The upper 90% supernatant was collected after centrifugation for the fabrication of the TFTs.^41^

*Deposition of Semiconducting CNT Channels*: The semiconducting CNT films were deposited on a PEN (Teijin DuPont Films; thickness, 125 µm) substrate by a dip‐coating method in a diluted CNT solution. First, the PEN substrate was treated with hexamethyl disilazane to guarantee a good surface wettability between CNTs and the substrate. Second, the substrate was immersed in the CNT solution heated by a water bath at 60 °C for different times to deposit uniform CNT networks with different CNT densities. Third, the substrate loaded with a semiconducting CNT film was washed in toluene, acetone, and isopropyl alcohol for 5 min successively. Finally, the sample was heated at 120 °C for 10 min after being blow‐dried with N_2_.

*Patterning Process by Using a Photosensitive Dry Film*: First, a photosensitive dry film (DuPont Riston MX9010; thickness, 10 µm) was laminated onto a PEN substrate by a roll‐to‐roll technique at a speed of 1.5 m min^−1^ under optimized conditions of 130 °C temperature and 1.0 bar pressure. Second, a 10 s UV exposure was applied to the dry film through a mask pattern and then a post‐treatment at 120 °C for 90 s was carried out to stabilize the pattern. Finally, the dry film pattern was formed after a developing process using a developer of 1.5% NaOH solution at 70 °C for 5 min.

*Device Fabrication of All‐CNT TFTs*: First, a mark (Ti/Au: 5/50 nm) was formed on a PEN substrate by photolithography, electron‐beam evaporation, and lift‐off processes to aid in the subsequent alignments of the transparent CNT films. The CNT film for the electrodes was transferred from a membrane filter to the substrate by a roll‐to‐roll transfer process. After lamination of the photosensitive dry film on the substrate, source/drain electrodes and interconnections were patterned by photolithography, developing and oxygen plasma etching processes. Next, the CNT film for the channels was deposited onto the substrate by immersion in a semiconducting CNT solution for 2 h and further patterned by the aforementioned method based on the photosensitive dry film. Subsequently, a 600‐nm‐thick insulator layer was formed onto the substrate by spin coating a layer of PMMA (Microchem, 950 kMW) on it and baking the substrate on a hotplate at 180 °C for 30 min. Due to the solubility of PMMA in alkaline solution during the developing process, the contact windows were formed by etching the PMMA insulator using acetone and a polydimethylglutarimide‐based resist mask.^2^ Finally, the CNT film for the gate electrodes was transferred onto the substrate and patterned using the same method as that used to pattern the source and drain layers.

*Characterization*: All electrical measurements were performed using a semiconductor analyzer (Agilent B1500A) under ambient conditions. The fabricated devices were characterized using an SEM (Nova NanoSEM 430, acceleration voltage of 1 kV) and an optical analysis instrument (Olympus BX51M). Optical transmittance was measured using a UV–vis–NIR spectroscopy (Cary 5000).

## Conflict of Interest

The authors declare no conflict of interest.

## Supporting information

Supplementary
