# Supplementary material for: High‐Throughput Fabrication of Flexible and Transparent All‐Carbon Nanotube Electronics
Source: Adv Sci (Weinh). 2018 Feb 20;5(5):1700965. doi: 10.1002/advs.201700965 (PMC5979759; doi:10.1002/advs.201700965)
Supplement: Supplementary file 1 — Supplementary [file ADVS-5-1700965-s001.pdf]

Copyright WILEY-VCH Verlag GmbH & Co. KGaA, 69469 Weinheim, Germany, 2018.

ADVANCED SCIENCE
Open Access

Supporting Information

for *Adv. Sci.,* DOI: 10.1002/advs.201700965

High-Throughput Fabrication of Flexible and Transparent All-Carbon Nanotube Electronics

Yong-Yang Chen, Yun Sun, Qian-Bing Zhu, Bing-Wei Wang, Xin Yan, Song Qiu,* Qing-Wen Li, Peng-Xiang Hou, Chang Liu, Dong-Ming Sun,* and Hui-Ming Cheng*

Supporting Information

High-throughput fabrication of flexible and transparent all-carbon nanotube electronics

Yong-Yang Chen, Yun Sun, Qian-Bing Zhu, Bing-Wei Wang, Xin Yan, Song Qiu*, Qing-Wen Li, Peng-Xiang Hou, Chang Liu, Dong-Ming Sun*, Hui-Ming Cheng*

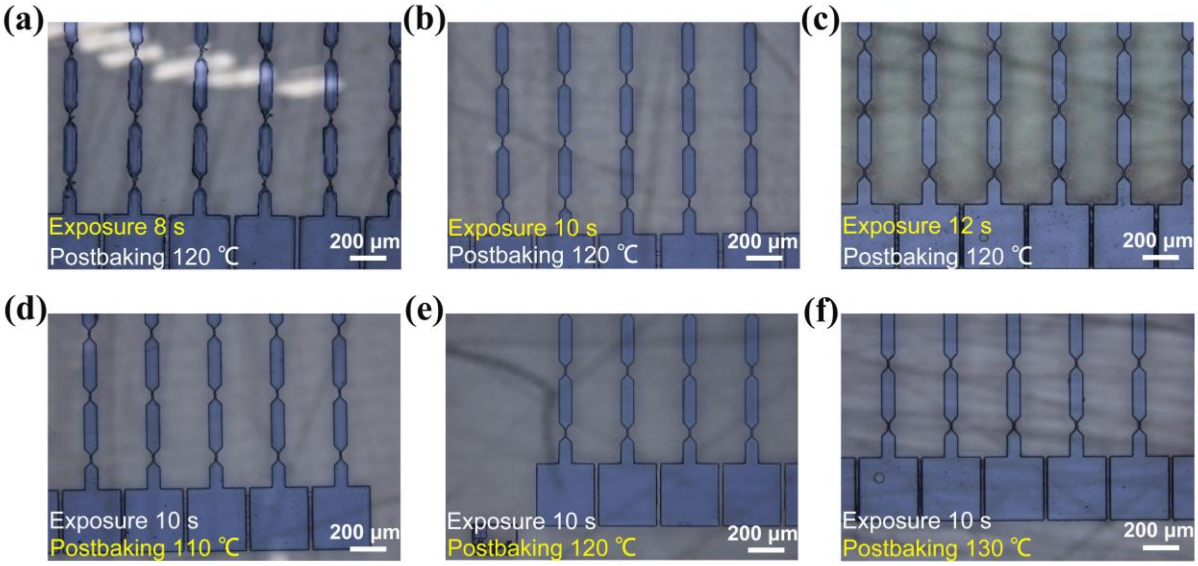

Figure S1. Optical images of the patterns obtained under various conditions of exposure time and baking temperature. Under the same baking condition of 120 ^{o}C for 90 s, the pattern obtained under an exposure time of 10 s (b) is better than those of 8 s (a) and 12 s (c). Under the same exposure time of 10 s, the pattern obtained under a baking temperature of 120 ^{o}C (e) is better than those of 110 ºC (d) and 130 ºC (f).

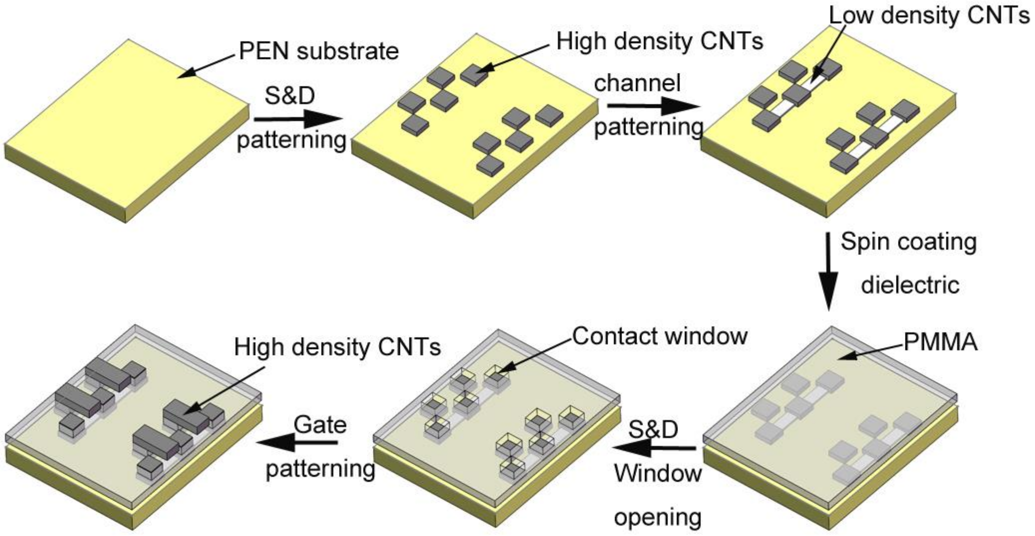

Figure S2. Fabrication processes of the split top-gate all-carbon TFTs and inverters on a PEN substrate. S&D represents source and drain electrode, respectively.

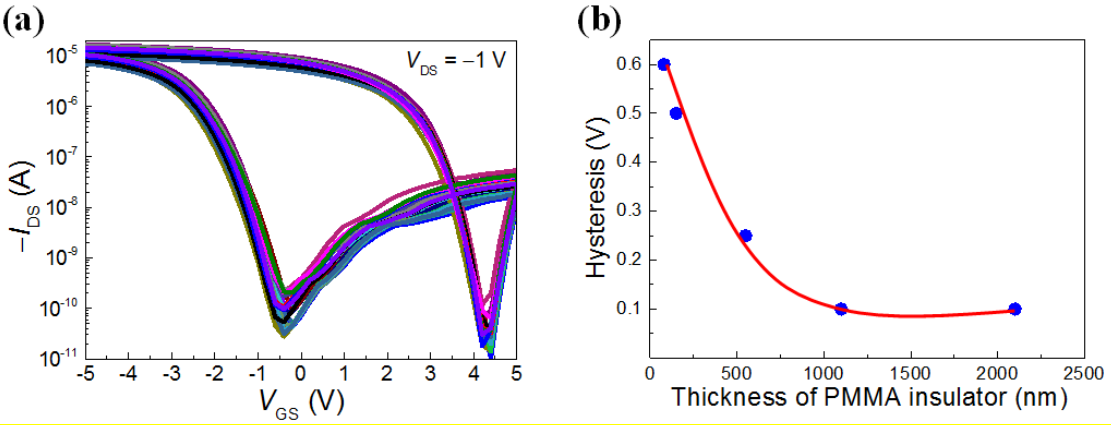

Figure S3. (a) Transfer characteristics of global bottom-gated CNT-TFTs. (b) Dependence of hysteresis and on the thickness of PMMA insulator.

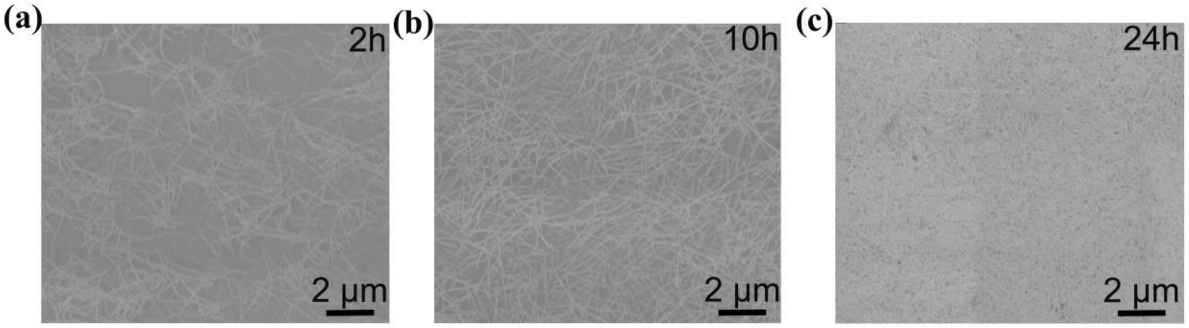

Figure S4. SEM images of the structures of semiconducting CNT films with deposition time of (a) 2 h, (b) 10 h, or (c) 24 h on a PEN substrate.

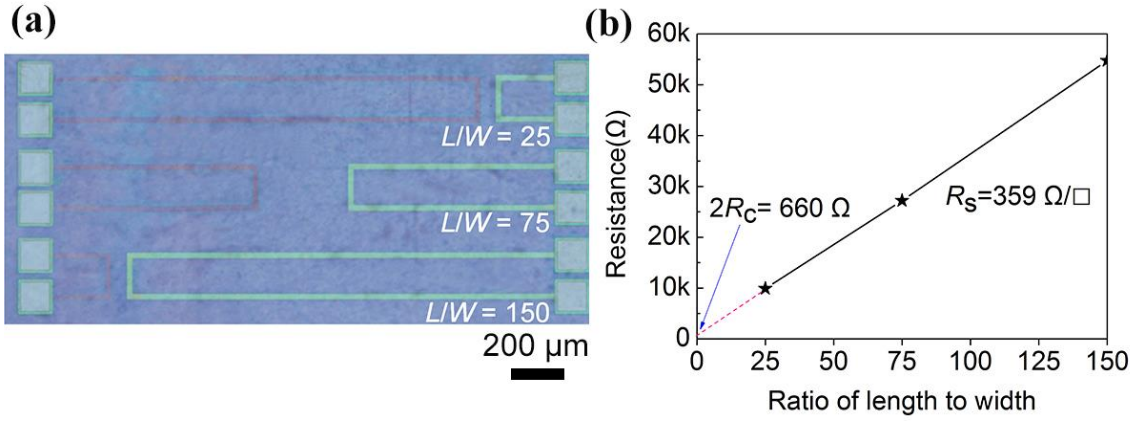

Figure S5. CNT films patterned by the photosensitive dry film patterning technique. a) Optical micrograph of CNT wires with different ratios of length to width (L/W). b) Sheet resistance of CNT thin films, where the slope corresponds to sheet resistance (R_S) and the intercept to the double contact resistance (R_C) between the CNT thin film and the probe.

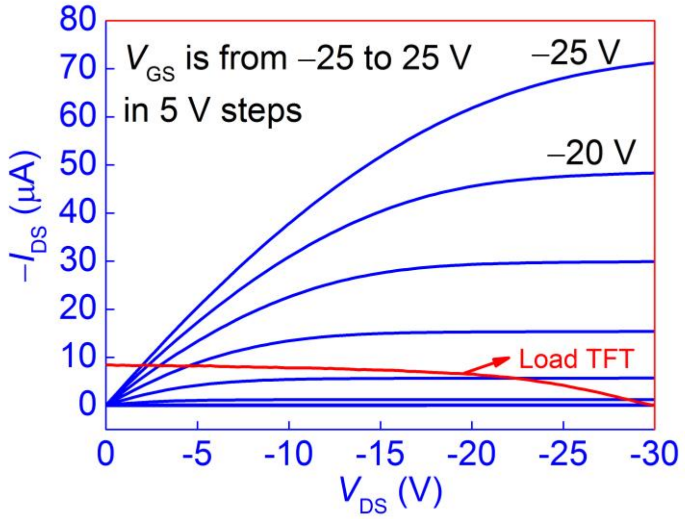

Figure S6. Output characteristics of a driver TFT and load TFT in a same all-CNT inverter.

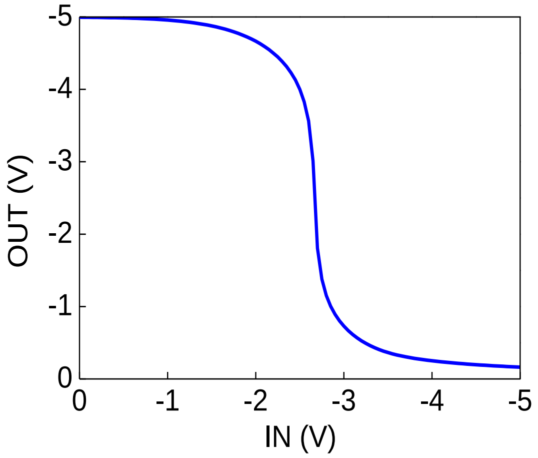

Figure S7. Transfer characteristics of an all-CNT inverter with a 50 nm Al_2O_3 layer as the gate dielectric.

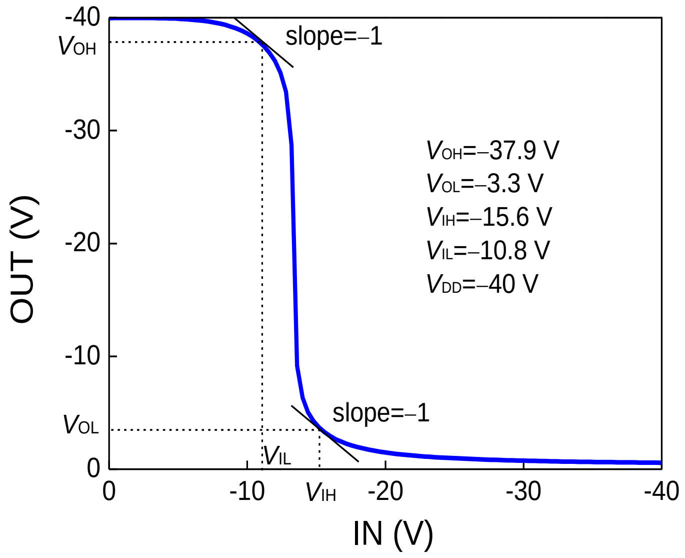

Figure S8. Definition of V_{OH}, V_{OL}, V_{IH} and V_{IL} based on the transfer characteristics of an inverter, where V_{OH}, V_{OL}, V_{IH} and V_{IL} corresponding the largest output voltage, smallest output voltage, highest input voltage and smallest input voltage where slope = –1, respectively.

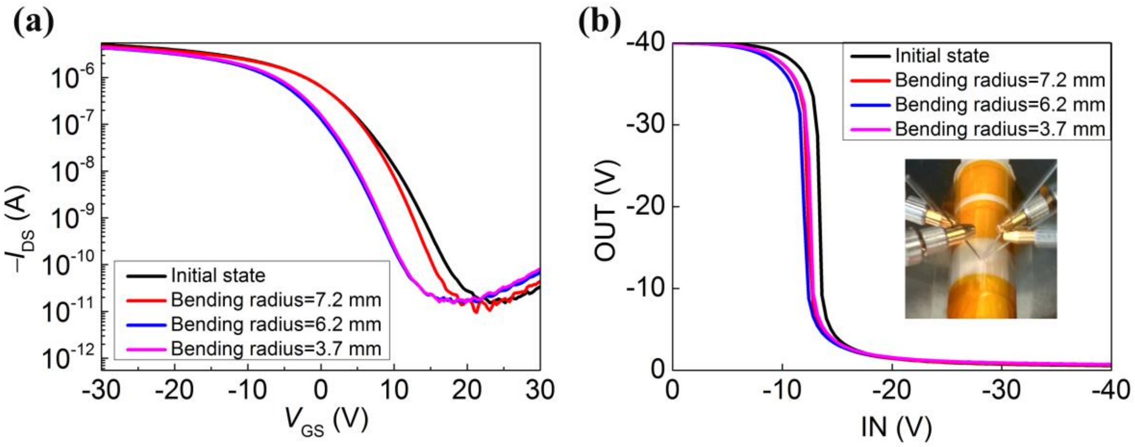

Figure S9. Transfer characteristics of an all-CNT TFT (a) and an inverter (b) bending at various radius. Inset of (b) is an actual image of the device prepared for the bending test.

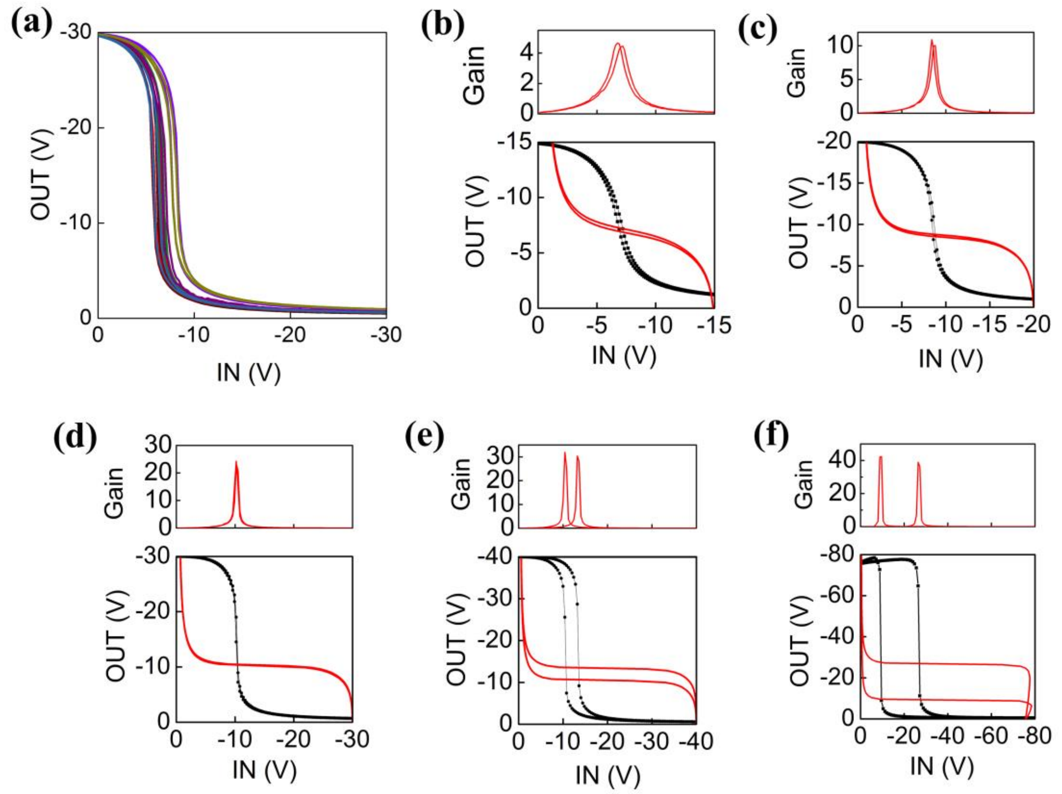

Figure S10. Electrical performance of all-CNT inverters. a) Input-output characteristics of 14 inverters showing good uniformity, V_{DD} = –30 V. b)-f), Input-output characteristics of an inverter under different operation voltages. Both the voltage gain and the hysteresis significantly increase with the increase of the operation voltage.
